# Supplementary material for: Burden of undiagnosed hypertension and its associated factors: A challenge for primary health care in urban Colombia
Source: PLoS One. 2023 Nov 28;18(11):e0294177. doi: 10.1371/journal.pone.0294177 (PMC10684019; doi:10.1371/journal.pone.0294177)
Supplement: S1 Checklist — (DOCX) [file pone.0294177.s001.docx]

**BURDEN OF UNDIAGNOSED HYPERTENSION AND ITS ASSOCIATED FACTORS: A CHALLENGE FOR PRIMARY HEALTH CARE IN URBAN COLOMBIA**

Jorge Emilio Salazar Flórez, Ángela Patricia Echeverri Rendón, Luz Stella Giraldo Cardona

STROBE Statement—checklist of items that should be included in reports of observational studies.

|  | Item No. | Recommendation | Page No. | Relevant text from manuscript |
| --- | --- | --- | --- | --- |
| **Title and abstract** | 1 | (*a*) Indicate the study’s design with a commonly used term in the title or the abstract | 3 | “Cross-sectional population-based study was conducted […]” |
|  |  | (*b*) Provide in the abstract an informative and balanced summary of what was done and what was found | 3,4 | “The prevalence of hypertension was estimated. The risk factors influencing the normotensive, diagnosed and undiagnosed hypertension were analyzed using multinomial regression. The outcome variable had three categories representing participants with normotensive (reference category), diagnosed, and undiagnosed hypertension. The model's multinomial regression coefficient was exponentiated and reported as relative risk ratios (RRR) with 95% confidence intervals (CI). This model adjusts for sex and sample weight for each neighborhood […]  The study revealed a hypertension prevalence of 38.5% and an undiagnosed hypertension rate of 50.9%. Those with undiagnosed hypertension were predominantly adults over 60 years (RRR= 0.68; 95% CI: 0.53- 0.86), individuals with an elementary school education (RRR=1.75; 95% CI: 1.27-2.42), those physically active (RRR= 1.52; 95% CI: 1.22-1.89), without prior diagnoses of chronic comorbidities (RRR= 1.42; 95% CI: 1.12-1.82), and with obesity (RRR=2.25; 95% CI: 1.63-3.11) or overweight conditions (RRR= 1.70; 95% CI: 1.334-2.15).” |
| Introduction | | | |  |
| Background/rationale | 2 | Explain the scientific background and rationale for the investigation being reported | 5-6 |  |
| Objectives | 3 | State specific objectives, including any prespecified hypotheses | 6 | “However, there is a distinct lack of data for Colombia and its neighbors, leading to the focus of this study, which aims to identify risk factors of undiagnosed hypertension in Colombian adults and guide policymakers in tailored strategy development” |
| Methods | | | |  |
| Study design | 4 | Present key elements of study design early in the paper | 6 | “A population-based cross-sectional study […]” |
| Setting | 5 | Describe the setting, locations, and relevant dates, including periods of recruitment, exposure, follow-up, and data collection | 6 | “Stratified sampling was applied according to three conditions: 1) area of residence: rural, urban; 2) age group: young adult (<41 years), adulthood (41-60 years) and older adult (>60 years); and 3) sex: male, female. To access the unit of analysis (adults over 18 years), a systematic random selection was applied in each neighborhood/ sidewalk with a selection criterion of every two households. The study was conducted from February 01, 2022 to December 20, 2022 in Sabaneta, Colombia.” |
| Participants | 6 | (*a*) *Cohort study*—Give the eligibility criteria, and the sources and methods of selection of participants. Describe methods of follow-up  *Case-control study*—Give the eligibility criteria, and the sources and methods of case ascertainment and control selection. Give the rationale for the choice of cases and controls  *Cross-sectional study*—Give the eligibility criteria, and the sources and methods of selection of participants | Not applicate |  |
|  |  | (*b*) *Cohort study*—For matched studies, give matching criteria and number of exposed and unexposed  *Case-control study*—For matched studies, give matching criteria and the number of controls per case | Not applicate |  |
| Variables | 7 | Clearly define all outcomes, exposures, predictors, potential confounders, and effect modifiers. Give diagnostic criteria, if applicable | 7-8 | […] “An individual was classified as hypertensive if they recorded a systolic blood pressure (SBP) of ≥140 mmHg and/or a diastolic blood pressure (DBP) of ≥90 mmHg, as per the JNC VI guidelines ^[24]^. Undiagnosed hypertension was characterized by having an SBP of ≥140 mmHg or a DBP of ≥90 mmHg, without any prior hypertension diagnosis from a healthcare professional or without being on antihypertensive medications. The study's primary variable of interest was segmented into three categories: normotensive, diagnosed hypertension, and undiagnosed hypertension.”  “[…] The World Health Organization (WHO) Chronic Diseases STEPwise approach to Surveillance (STEPS) survey was incorporated, designed to assess behavioral, anthropometric, biological, and demographic attributes ^[27]^. Pertinent to our study, we extracted demographic details such as gender, age, marital status, and education level. Behavioral risk factors considered included smoking, alcohol consumption, and fruit and vegetable intake. Cardiometabolic risk factors taken into account were high body mass index, diabetes, and elevated cholesterol.  Additionally, the Pérez-Rojas-García test for classifying sedentary lifestyles was administered ^[28]^. Participants were stratified as either severely sedentary, moderately sedentary, active, or highly active based on the test outcomes. […]”. |
| Data sources/ measurement | 8* | For each variable of interest, give sources of data and details of methods of assessment (measurement). Describe comparability of assessment methods if there is more than one group | 7-8 | “[…] Vital signs of all participants were checked three times. Heart rate was determined using an oximeter (model MD300C29) after a 15-minute rest. Arterial tension was assessed using a pre-calibrated manometer (model CE 0297). The procedure for measuring arterial pressure adhered to the guidelines of the Sixth Joint National Committee (JNC VI) ^[24]^ which is in line with the recommendations of the American Heart Association ^[25]^, the American Society of Hypertension, and the Pan American Health Organization (PAHO) ^[26]^ […]”  “[…] Anthropometric measurements were taken using various instruments. A calibrated balance model 142KL was used for weight determinations, and for height, a tallimeter with a movable foot was employed. Abdominal and pelvic circumferences were measured with a handheld tape measure. […]”.  “[…] The World Health Organization (WHO) Chronic Diseases STEPwise approach to Surveillance (STEPS) survey was incorporated, designed to assess behavioral, anthropometric, biological, and demographic attributes ^[27]^ […]”  “[…] Additionally, the Pérez-Rojas-García test for classifying sedentary lifestyles was administered ^[28]^. Participants were stratified as either severely sedentary, moderately sedentary, active, or highly active based on the test outcomes. […]” |
| Bias | 9 | Describe any efforts to address potential sources of bias | 21-22 | “[…] Firstly, due to the cross-sectional design, it wasn't possible to infer causality. Secondly, relying on self-reported data, such as diagnosed hypertension, might introduce recall bias. Thirdly, family history was not factored in as a covariate. Fourthly, blood pressure in the study was measured three times during physical examinations, deviating from the Hypertension Clinical Practice Guidelines that recommend an average of ≥2 readings obtained on ≥2 separate occasions ^[25]^. Fifthly, although the desired sample size was not achieved, it is noteworthy that the attrition rate stood at a modest 6.5% (n=20). Additionally, considering the actual hypertension prevalence observed in this study (38.5%), the precision amounted to 5.6. Thus, the concluding sample of 286 participants did not markedly compromise the estimate's accuracy, an essential metric in prevalence research. […]”  “[…] Regardless of these constraints, our study carved a niche for itself. It utilized a population-based sample that mirrors the adult demographic across the municipality, meticulously analyzed the risk factors tied to undiagnosed hypertension, and furnished insights to guide forthcoming hypertension prevention initiatives […]” |
| Study size | 10 | Explain how the study size was arrived at | 6-7 | “To determine the sample size, we referenced the 2019 population projection from the National Administrative Department of Statistics (DANE, as per its Spanish acronym). In 2019, the population aged over 18 in the municipality totaled 69,045 individuals ^[23]^. A formula was utilized to project a prevalence, setting a 95.0% confidence interval, an anticipated hypertension prevalence of 28.0%, and a maximum permissible error of 5.0%. This resulted in a targeted sample of 306 individuals. The computation was executed using the free online software, Open Epi 3.01. The study ultimately encompassed 286 adults, translating to a response rate of 93.5%.” |

| Quantitative variables | | 11 | | Explain how quantitative variables were handled in the analyses. If applicable, describe which groupings were chosen and why | 9 | “[…] while means and standard deviations described the quantitative variables.” | |
| --- | --- | --- | --- | --- | --- | --- | --- |
| Statistical methods | | 12 | | (*a*) Describe all statistical methods, including those used to control for confounding | 9-10 | “[…] Data analysis was conducted using R software (v 4.2.2, www.r-project.org/). The demographic attributes, lifestyle habits, anthropometric indices, and comorbidities were presented through absolute frequencies and percentages for qualitative variables, while means and standard deviations described the quantitative variables. Data was segmented based on three categories: normotensive, diagnosed hypertension, and undiagnosed hypertension. Differences across these categories in terms of general characteristics, lifestyle, and comorbidities were assessed via the chi-square test of independence. All statistical tests were two-tailed with a significance level set at α= 0.05.  For understanding the relationship between dependent and independent variables, a multivariable multinomial regression model was employed, adjusting for potential confounders. Every variable was introduced into the multinomial logistic regression model in one step, and the least significant ones were sequentially removed until the most concise model was achieved. The dependent variable had three classifications: normotensive, diagnosed hypertension, and undiagnosed hypertension, with the normotensive group serving as the reference for comparison in the regression analysis. Coefficients from the multinomial regression were exponentiated and presented as relative risk ratios (RRR) with their corresponding 95% confidence intervals (CI). Here, the RRR represents the likelihood of an outcome in the exposed group relative to its occurrence in the unexposed group. In the context of this study, a p-value less than 0.05 indicated statistical significance. Independent variables encompassed aspects like lifestyle choices, substance use, dietary habits, anthropometric indicators, sedentary behavior, and existing comorbidities. Adjustments in this model were made for gender and a weighting variable representing the distribution of participation across neighborhoods. The choice of variables incorporated in the multinomial model was driven by their theoretical relevance, as depicted in figure 1.” | |
|  |  |  |  | (*b*) Describe any methods used to examine subgroups and interactions | Not applicate |  | |
|  |  |  |  | (*c*) Explain how missing data were addressed | Not applicate |  | |
|  |  |  |  | (*d*) *Cohort study*—If applicable, explain how loss to follow-up was addressed  *Case-control study*—If applicable, explain how matching of cases and controls was addressed  *Cross-sectional study*—If applicable, describe analytical methods taking account of sampling strategy | Not applicate |  | |
|  |  |  |  | (*e*) Describe any sensitivity analyses | Not applicate |  | |
| Results | | | | | | | |
| Participants | | 13* | | (a) Report numbers of individuals at each stage of study—eg numbers potentially eligible, examined for eligibility, confirmed eligible, included in the study, completing follow-up, and analysed | 7 | “[…] This resulted in a targeted sample of 306 individuals. The computation was executed using the free online software, Open Epi 3.01. The study ultimately encompassed 286 adults, translating to a response rate of 93.5%. […] ” | |
|  |  |  |  | (b) Give reasons for non-participation at each stage | 7 | “[…] The study ultimately encompassed 286 adults, translating to a response rate of 93.5%. […] ” | |
|  |  |  |  | (c) Consider use of a flow diagram | Not applicate |  | |
| Descriptive data | | 14* | | (a) Give characteristics of study participants (eg demographic, clinical, social) and information on exposures and potential confounders | 12-14 | Table 1, Table 2 | |
|  |  |  |  | (b) Indicate number of participants with missing data for each variable of interest | Not applicate |  | |
|  |  |  |  | (c) *Cohort study*—Summarise follow-up time (eg, average and total amount) | Not applicate |  | |
| Outcome data | | 15* | | *Cohort study*—Report numbers of outcome events or summary measures over time | Not applicate |  | |
|  |  |  |  | *Case-control study—*Report numbers in each exposure category, or summary measures of exposure | Not applicate |  | |
|  |  |  |  | *Cross-sectional study—*Report numbers of outcome events or summary measures | 12-15 | Table 1, Table 2 and Table 3, figure 2 | |
| Main results | | 16 | | (*a*) Give unadjusted estimates and, if applicable, confounder-adjusted estimates and their precision (eg, 95% confidence interval). Make clear which confounders were adjusted for and why they were included | 12-15 | Table 1, Table 2 and Table 3, figure 2 | |
|  |  |  |  | (*b*) Report category boundaries when continuous variables were categorized | 7-8 | “An individual was classified as hypertensive if they recorded a systolic blood pressure (SBP) of ≥140 mmHg and/or a diastolic blood pressure (DBP) of ≥90 mmHg, as per the JNC VI guidelines ^[24]^. Undiagnosed hypertension was characterized by having an SBP of ≥140 mmHg or a DBP of ≥90 mmHg, without any prior hypertension diagnosis from a healthcare professional or without being on antihypertensive medications. The study's primary variable of interest was segmented into three categories: normotensive, diagnosed hypertension, and undiagnosed hypertension […].  “[…] Additionally, the Pérez-Rojas-García test for classifying sedentary lifestyles was administered ^[28]^. Participants were stratified as either severely sedentary, moderately sedentary, active, or highly active based on the test outcomes.” | |
|  |  |  |  | (*c*) If relevant, consider translating estimates of relative risk into absolute risk for a meaningful time period | Not applicate |  | |
| Other analyses | 17 | | Report other analyses done—eg analyses of subgroups and interactions, and sensitivity analyses | | Not applicate | |  |
| Discussion | | | | | | | |
| Key results | 18 | | Summarise key results with reference to study objectives | | 16 | | “The study aimed to estimate the prevalence of undiagnosed hypertension and its associated factors in individuals aged 18 and above in a Colombian municipality. The prevalence rates for hypertension and undiagnosed hypertension stood at 38.5% and 50.9%, respectively. In the present study, individuals who had completed either elementary or high school, had no chronic diseases, were physically active, and were either obesity or overweight were more inclined to be unaware of their hypertensive status.” |
| Limitations | 19 | | Discuss limitations of the study, taking into account sources of potential bias or imprecision. Discuss both direction and magnitude of any potential bias | | 21 | | “The study presented several limitations. Firstly, due to the cross-sectional design, it wasn't possible to infer causality. Secondly, relying on self-reported data, such as diagnosed hypertension, might introduce recall bias. Thirdly, family history was not factored in as a covariate. Fourthly, blood pressure in the study was measured three times during physical examinations, deviating from the Hypertension Clinical Practice Guidelines that recommend an average of ≥2 readings obtained on ≥2 separate occasions ^[25]^. Fifthly, although the desired sample size was not achieved, it is noteworthy that the attrition rate stood at a modest 6.5% (n=20). Additionally, considering the actual hypertension prevalence observed in this study (38.5%), the precision amounted to 5.6. Thus, the concluding sample of 286 participants did not markedly compromise the estimate's accuracy, an essential metric in prevalence research.” |
| Interpretation | 20 | | Give a cautious overall interpretation of results considering objectives, limitations, multiplicity of analyses, results from similar studies, and other relevant evidence | | 16-22 | |  |
| Generalisability | 21 | | Discuss the generalisability (external validity) of the study results | | 16-22 | |  |
| Other information | | |  | | | | |
| Funding | 22 | | Give the source of funding and the role of the funders for the present study and, if applicable, for the original study on which the present article is based | | 22 | | “This study was funded by the San Martin University Foundation (PY-2020-025).” |

*Give information separately for cases and controls in case-control studies and, if applicable, for exposed and unexposed groups in cohort and cross-sectional studies.

**Note:** An Explanation and Elaboration article discusses each checklist item and gives methodological background and published examples of transparent reporting. The STROBE checklist is best used in conjunction with this article (freely available on the Web sites of PLoS Medicine at http://www.plosmedicine.org/, Annals of Internal Medicine at http://www.annals.org/, and Epidemiology at http://www.epidem.com/). Information on the STROBE Initiative is available at www.strobe-statement.org.
