# Supplementary material for: Burden of undiagnosed hypertension and its associated factors: A challenge for primary health care in urban Colombia
Source: PLoS One. 2023 Nov 28;18(11):e0294177. doi: 10.1371/journal.pone.0294177 (PMC10684019; doi:10.1371/journal.pone.0294177)
Supplement: Attachment — Submitted filename: PONE-D-23-08509_reviewer_dm.pdf [file pone.0294177.s002.pdf]

PLOS ONE

BURDEN OF UNDIAGNOSTIC HYPERTENSION ANN ASSOCIATED FACTORS: A CHALLENGE FOR PRIMARY HEALTH CARE

--Manuscript Draft--

**Manuscript Number:**	PONE-D-23-08509
**Article Type:**	Research Article
**Full Title:**	BURDEN OF UNDIAGNOSTIC HYPERTENSION ANN ASSOCIATED FACTORS: A CHALLENGE FOR PRIMARY HEALTH CARE
**Short Title:**	BURDEN OF UNDIAGNOSTIC HYPERTENSION ANN ASSOCIATED FACTORS
**Corresponding Author:**	Jorge Emilio Salazar Flórez<br>Fundacion Universitaria San Martin<br>Sabaneta, COLOMBIA
**Keywords:**	Hypertension, Risk Factors, Early Diagnosis, Primary Health Care, Chronic Disease
**Abstract:**	Background: Arterial hypertension is one of the most prevalent chronic non-communicable diseases. It is the leading preventable risk factor for cardiovascular disease (CVD) and all-cause mortality worldwide. Its leading causes and consequences are preventable, yet it remains an undiagnosed opportunity. Consequently, the aim is to determine the prevalence and factors associated with unscreened hypertension in adults.Methods and Findings: Cross-sectional population-based study was conducted between 2021-2022 with 286 older than 18 years, using stratified and systematic random sampling. The World Health Organization STEP survey and the Perez Rojas test were applied to assess risk behaviors and sedentary lifestyles. Body mass index, waist circumference, and arterial tension were evaluated using standardized instruments. The prevalence of hypertension was estimated. The risk factors influencing the unscreened hypertension were analyzed using a Generalized Linear Model (GLM) by the Poisson family and log link. This model adjusts for sex and sample weight for each neighborhood. We estimate relative risks with a confidence interval of 95 %. A p-value of lower than 0.05 was statistically significant. We found a prevalence of hypertension of 38.5% and a burden of unscreened hypertension of 50.9%. People with unscreened hypertension typically were younger than 65 years (aRR= 2.28; 95% CI: 1.7- 3.1), physically active (aRR= 2.1; 95% CI: 1.6-2.6), had no previous diagnoses of chronic comorbidities (aRR= 2.0; 1 95% CI: 1.6-2.8), didn't consume alcohol in the last year (aRR= 1.3; 95% CI: 1.1-1.7) and with normal abdominal conditions of weight (aRR= 1.4; 95% CI: 1.1-1.8). Conclusions: Unscreened hypertension was notable in the population without risk conditions. Community-based early detection and education strategies are needed to prevent this problem.
**Order of Authors:**	Jorge Emilio Salazar Flórez
	Ángela Patricia Echeverri Rendón
	Luz Stella Giraldo Cardona
**Additional Information:**	
**Question**	**Response**
**Financial Disclosure**<br><br>Enter a financial disclosure statement that describes the sources of funding for the work included in this submission. Review the submission guidelines for detailed requirements. View published research articles from *PLOS ONE* for specific examples.	The author(s) received no specific funding for this work.

Powered by Editorial Manager® and ProduXion Manager® from Aries Systems Corporation

This statement is required for submission and **will appear in the published article** if the submission is accepted. Please make sure it is accurate.<br><br>**Unfunded studies**<br>Enter: *The author(s) received no specific funding for this work.*<br><br>**Funded studies**<br>Enter a statement with the following details:<br>• Initials of the authors who received each award<br>• Grant numbers awarded to each author<br>• The full name of each funder<br>• URL of each funder website<br>• Did the sponsors or funders play any role in the study design, data collection and analysis, decision to publish, or preparation of the manuscript?<br>• **NO** - Include this sentence at the end of your statement: *The funders had no role in study design, data collection and analysis, decision to publish, or preparation of the manuscript.*<br>• **YES** - Specify the role(s) played.<br><br>* typeset	
**Competing Interests**<br><br>Use the instructions below to enter a competing interest statement for this submission. On behalf of all authors, disclose any competing interests that could be perceived to bias this work—acknowledging all financial support and any other relevant financial or non-financial competing interests.<br><br>This statement is required for submission and **will appear in the published article** if the submission is accepted. Please make sure it is accurate and that any funding sources listed in your Funding Information later in the submission form are also declared in your Financial Disclosure statement.<br><br>View published research articles from *PLOS ONE* for specific examples.	The authors have declared that no competing interests exist.

**NO authors have competing interests**<br><br>Enter: *The authors have declared that no competing interests exist.*<br><br>**Authors with competing interests**<br><br>Enter competing interest details beginning with this statement:<br><br>*I have read the journal's policy and the authors of this manuscript have the following competing interests: [insert competing interests here]*<br><br>* typeset	
**Ethics Statement**<br><br>Enter an ethics statement for this submission. This statement is required if the study involved:<br><br>• Human participants<br>• Human specimens or tissue<br>• Vertebrate animals or cephalopods<br>• Vertebrate embryos or tissues<br>• Field research<br><br>Write "N/A" if the submission does not require an ethics statement.<br><br>General guidance is provided below. Consult the submission guidelines for detailed instructions. **Make sure that all information entered here is included in the Methods section of the manuscript.**	The University of San Martín research committee approved the study under the 016-2018 code. All participants signed the informed consent.

**Format for specific study types**<br><br>**Human Subject Research (involving human participants and/or tissue)**<br>• Give the name of the institutional review board or ethics committee that approved the study<br>• Include the approval number and/or a statement indicating approval of this research<br>• Indicate the form of consent obtained (written/oral) or the reason that consent was not obtained (e.g. the data were analyzed anonymously)<br><br>**Animal Research (involving vertebrate animals, embryos or tissues)**<br>• Provide the name of the Institutional Animal Care and Use Committee (IACUC) or other relevant ethics board that reviewed the study protocol, and indicate whether they approved this research or granted a formal waiver of ethical approval<br>• Include an approval number if one was obtained<br>• If the study involved *non-human primates*, add *additional details* about animal welfare and steps taken to ameliorate suffering<br>• If anesthesia, euthanasia, or any kind of animal sacrifice is part of the study, include briefly which substances and/or methods were applied<br><br>**Field Research**<br><br>Include the following details if this study involves the collection of plant, animal, or other materials from a natural setting:<br>• Field permit number<br>• Name of the institution or relevant body that granted permission	
**Data Availability**<br><br>Authors are required to make all data underlying the findings described fully available, without restriction, and from the time of publication. PLOS allows rare exceptions to address legal and ethical concerns. See the PLOS Data Policy and FAQ for detailed information.	Yes - all data are fully available without restriction

A Data Availability Statement describing where the data can be found is required at submission. Your answers to this question constitute the Data Availability Statement and **will be published in the article**, if accepted.<br><br>**Important:** Stating 'data available on request from the author' is not sufficient. If your data are only available upon request, select 'No' for the first question and explain your exceptional situation in the text box.<br><br>Do the authors confirm that all data underlying the findings described in their manuscript are fully available without restriction?	
**Describe where the data may be found in full sentences. If you are copying our sample text, replace any instances of XXX with the appropriate details.**<br><br>• If the data are **held or will be held in a public repository**, include URLs, accession numbers or DOIs. If this information will only be available after acceptance, indicate this by ticking the box below. For example: *All XXX files are available from the XXX database (accession number(s) XXX, XXX.).*<br>• If the data are all contained **within the manuscript and/or Supporting Information files**, enter the following: *All relevant data are within the manuscript and its Supporting Information files.*<br>• If neither of these applies but you are able to provide **details of access elsewhere**, with or without limitations, please do so. For example:<br><br>*Data cannot be shared publicly because of [XXX]. Data are available from the XXX Institutional Data Access / Ethics Committee (contact via XXX) for researchers who meet the criteria for access to confidential data.*<br><br>*The data underlying the results presented in the study are available from (include the name of the third party*	All relevant data are within the manuscript and its Supporting Information files

and contact information or URL).<br>• This text is appropriate if the data are owned by a third party and authors do not have permission to share the data.<br><br>* typeset	
Additional data availability information:	

Manuscript
Click here to access/download;Manuscript;Paper HTA PLOS.docx

BURDEN OF UNDIAGNOSTIC HYPERTENSION ANN ASSOCIATED FACTORS: A CHALLENGE FOR PRIMARY HEALTH CARE

BURDEN OF UNDIAGNOSTIC HYPERTENSION ANN ASSOCIATED FACTORS

Jorge Emilio Salazar Flórez[1], Ángela Patricia Echeverri Rendón[2], Luz Stella Giraldo Cardona[3]

1. Research Center, Grupo de Estudio de Enfermedades Infecciosas y Crónicas (GEINCRO), San Martin University Foundation, Colombia. CES University, group of epidemiology and biostatistics. ORCID: 0000-0002-7666-9099. Email: jorge.salazarf@sanmartin.edu.co
2. Grupo de Estudio de Enfermedades Infecciosas y Crónicas (GEINCRO), San Martin University Foundation, Colombia. ORCID: 0000-0001-9441-7381. Email: 30211182032@est.sanmartin.edu.co
3. Research Center, Grupo de Estudio de Enfermedades Infecciosas y Crónicas (GEINCRO), San Martin University Foundation, Colombia. ORCID: 0000-0003-0192-2798. Email: luz.giraldo@sanmartin.edu.co

Correspondence:

Jorge Emilio Salazar Flórez. Address: Calle 75 sur # 34-50, Block 2. Faculty of Medicine, San Martin University Foundation, Sabaneta, Antioquia, Colombia. Phone: 604-5906983 - 5025. email: jorge.salazarf@sanmartin.edu.co

Abstract

Background: Arterial hypertension is one of the most prevalent chronic non-communicable diseases. It is the leading preventable risk factor for cardiovascular disease (CVD) and all-cause mortality worldwide. Its leading causes and consequences are preventable, yet it remains an undiagnosed opportunity. Consequently, the aim is to determine the prevalence and factors associated with unscreened hypertension in adults.

Methods and Findings: Cross-sectional population-based study was conducted between 2021-2022 with 286 older than 18 years, using stratified and systematic random sampling. The World Health Organization STEP survey and the Perez Rojas test were applied to assess risk behaviors and sedentary lifestyles. Body mass index, waist circumference, and arterial tension were evaluated using standardized instruments. The prevalence of hypertension was estimated. The risk factors influencing the unscreened hypertension were analyzed using a Generalized Linear Model (GLM) by the Poisson family and log link. This model adjusts for sex and sample weight for each neighborhood. We estimate relative risks with a confidence interval of 95 %. A p-value of lower than 0.05 was statistically significant. We found a prevalence of hypertension of 38.5% and a burden of unscreened hypertension of 50.9%. People with unscreened hypertension typically were younger than 65 years (aRR= 2.28; 95% CI: 1.7- 3.1), physically active (aRR= 2.1; 95% CI: 1.6-2.6), had no previous diagnoses of chronic comorbidities (aRR= 2.0; 1 95% CI: 1.6-2.8), didn't consume alcohol in the last year (aRR= 1.3; 95% CI: 1.1-1.7) and with normal abdominal conditions of weight (aRR= 1.4; 95% CI: 1.1-1.8).

Conclusions: Unscreened hypertension was notable in the population without risk conditions. Community-based early detection and education strategies are needed to prevent this problem.

Keywords: Hypertension, Risk Factors, Early Diagnosis, Primary Health Care, Chronic Disease

Introduction

Arterial hypertension is one of the most prevalent chronic noncommunicable diseases [1]. It is the principal preventable risk factor for cardiovascular disease (CVD) and all-cause mortality worldwide [2]. Raised Blood Pressure (BP) is defined as systolic blood pressure (SBP) ≥140 mmHg or diastolic blood pressure (DBP) ≥90 mmHg. Hypertension (HTN) is responsible for millions of deaths globally, accounting for 10.4 million deaths in 2017 [1, 2] and years lived with disability. Hypertension affects more than 20.0% of the world's adult population [3]. This pathology is determined by multiple factors, both genetic, social, and environmental, including sex, age, use of psychoactive substances, alcohol, cigarettes, sedentary lifestyle, healthcare access, such as obesity, unhealthy eating through high sodium intake, and low consumption of fruits and vegetables [1, 4, 5, 6, 7].

Additionally, hypertension and chronic diseases represent a substantial financial cost. The global financial burden of high BP in 2001 was estimated to be about 10.0% of the world's total health costs [8]. Supporting a primary health care program for hypertension in Colombia amounts to 184,631 Colombian pesos per patient monthly. If the patient develops a complication (e.g., hemodialysis), the costs can increase from 780,000 pesos to 4,000,000 million COP annually [9].

In low- and middle-income countries, the prevalence of HTN is higher and the frequency of diagnosis, treatment, and control are lower than observed in high-income countries [1, 5]. Of the hypertensive individuals, 75.0% are from low-income and middle-income countries (LMICs) [1, 6]. A review that analyzed data from 844 studies performed in 154 countries with 8.69 million participants estimated that in the last 40 years, high-income countries experienced a significant BP decrease, while BP in LMIC regions increased [7]. It is known

that most Latin American countries are LMICs, consequently, with a high burden of disease and mortality from hypertension and chronic diseases [10]. CVD is the leading cause of death and disability in the Americas. In 2019, two million lives were lost, representing one-third of all regional deaths. For the same year, 34.4% of the population in the Americas had HTN, being controlled in only 40.9% of women and 32.2% of men [11, 12, 13]. However, most people with hypertension are asymptomatic. This leads to a high rate of undiagnosed hypertension.

In effect, adequate diagnosis, treatment, and control of hypertension continue to be a challenge in reducing the incidence of premature mortality and disability due to CVD, which is a reality in all countries of the world [11, 12, 14]. The main challenges continue to be early diagnosis and adherence to treatment. Approximately 580 million people with HTN (46%) are unaware of their condition because they have never been diagnosed [15]. A recent study that screened adults aged 15-54 indicated that 66.8% (n=6886) had never been diagnosed [16].

Undiagnosed and consequently untreated arterial hypertension is potentially fatal and can cause multiple complications. If HTN remains undiagnosed and untreated, serious health consequences are indicated, including cardiovascular morbidity and mortality [10, 11, 13]. Poor diagnosis of hypertension starts a cascade of risks. Dhungana et al. [4] refer to this as the gap in the hypertension care cascade: unscreened, unaware, untreated, uncontrolled; consequently, high health risks.

There is a high prevalence of undiagnosed hypertension, ranging from 50.4% to 59.9%, mainly in Asian or African countries [14, 15, 17, 18, 19]. Recently, conditions related to undiagnosed hypertension have been explored in some regions. An association has been found with male sex, current tobacco use, and high physical activity; additionally, overweight

respondents exhibit an increased likelihood of having undiagnosed and known hypertension [17, 18, 19].

However, few studies explore the factors associated with gaps in the cascade of hypertension care or studies that explore the factors related to the burden of undiagnosed hypertension. In the Americas, the most up-to-date information dates to 2010, with the Cardiovascular Risk Factor Multiple Evaluation in Latin America (CARMELA) study, which reported the prevalence of undiagnosed hypertension varying between 24.0% and 47.0% in seven cities of Latin America [20]. The nearest investigations were conducted in the United States in 2018, with a prevalence's undiagnosed HTN of less than 7.0% [21]. In South America, a study in Peru in 2021 reported a prevalence of undiagnosed hypertension of 67.0% among 3,697 hypertensive patients [22]. There is no updated data on the prevalence and associated factors of undiagnosed HTN in the general adult population in Colombia. The situation is similar in other countries. Therefore, this study aimed to examine risk factors associated with undiagnostic HTN among people over 18 in a Colombian municipality. Health policymakers might require such empirical evidence to create targeted strategies for controlling HTN in the country and the world.

Materials and methods

Population-based cross-sectional study. The reporting of the results followed the guidelines of the STROBE for observational study (supplementary 1). Stratified sampling was applied according to three conditions: 1) area of residence: rural, urban; 2) age group: young adult (<41 years), adulthood (41-60 years) and older adult (>60 years); and 3) sex: male, female. To access the unit of analysis (adults over 18 years), a systematic random selection was applied in each neighborhood/sidewalk with a selection criterion of every two households. The study was conducted from February 01, 2022 to December 20, 2022.

For the calculation of the sample, the population projection of the National Administrative Department of Statistics (DANE, acronym in Spanish) of 2019 was a reference. In 2019, the available universe of the population older than 18 for the municipality was 19,778 subjects. The formula was applied to estimate a prevalence, establishing a confidence interval of 95.0%, an expected prevalence of hypertension of 28.0%, and a maximum permissible error of 5.0%. A required sample of 306 individuals was obtained. The calculation was estimated in the free online software Open Epi 3.01. The final participation of 286 adults was recorded, representing a response rate of 93.5 %.

Instruments

Data collection was face-to-face and conducted by medical students qualified and experienced to conduct the work. Demographic data, anthropometric indicators, sedentary lifestyle, risk behaviors, arterial and heart frequency.

All participants had their vital signs checked three times. Heart frequency was determined using an oximeter model MD300C29 after 15 minutes of inactivity. The arterial tension was

evaluated with a pre-calibrated manometer model CE 0297. The measure of arterial pressure followed the recommendations by the Sixth Joint Committee National (JNC VI) [23], based on the recommendations of the American Heart Association [24], the American Society of Hypertension, and the Panamerican Health Organization (PAHO) [25]. Measurement was taken after 15 minutes of repose. The enumerators recorded three systolic and diastolic blood pressure readings at five-minute intervals. An adult was considered hypertensive if he/she had a systolic blood pressure (SBP) ≥140 mmHg and/or diastolic blood pressure (DBP) ≥ 90 mmHg according to JNC VI recommendations [23]. Undiagnosed hypertension was defined as having SBP ≥ 140 mmHg or DBP ≥ 90 mmHg and having never been told by a doctor or other health worker that they had raised blood pressure or hypertension and had not been taking antihypertensive medication.

Anthropometric measurements were also taken. A calibrated balance model 142KL was used for weight measurements. For height measurements, a tallimeter with a movable foot was used. Abdominal and pelvic circumferences were taken with a hand meter.

The survey used the World Health Organization (WHO) Chronic Diseases STEPwise approach to Surveillance (STEPS) survey, structured to measure the behavioral, anthropometric, biological, and demographic characteristics [26]. For our study purpose, we use the demographic (gender, age, marital status, and education level), behavioral risk factors (smoking, alcohol use, fruit and vegetable intake), and cardiometabolic risk factors (high body mass index, diabetes, and high cholesterol).

Finally, the Pérez-Rojas-García sedentary lifestyle classification test was administered [27]. This test categorized the person as severely sedentary, moderately sedentary, active, or very active. First, the heart frequency was taken; subsequently, the person was exposed to three

loads of work with different intensities. The first load had a beat rate of 68 beats, the second 104 beats, and the third 144 beats. The test consisted of ascending and descending a step with a height of 25 cm for 3 minutes per load with 1-minute rest intervals. If, after the first load, the heart frequency value remained lower than 120 beats/minute (p/m), the second load was applied, and so on, until completing the three loads. The test ended if the heart frequency was higher than 120 bpm during the first load, and the classification was severely sedentary. The patient was classified as moderately sedentary if the second load was completed. The instrument was applied to the Colombian population between 18 and 60 years of age [28].

Statistical Analysis

Data analysis was performed in R (v 4.2.2, www.r-project.org/). We described demographic characteristics, lifestyle, anthropometric index, and comorbidity using absolute frequencies and percentages for qualitative variables; and means and standard deviations for quantitative variables. We analyzed the arterial tension differences according to their general characteristics, lifestyle, and comorbidity using the chi-square of independence. A two-tailed significance level of $\alpha = 0.05$ was adopted for all statistical analyses.

Finally, the magnitude of the association between risk factors, and the incidence of unscreened hypertension was estimated based on the risk ratios or relative risk (RR), and the respective 95% confidence intervals (95% CI). These risk ratios were analyzed using a multivariate model using a Generalized Linear Model (GLM) on the data by the maximum likelihood method, with the Poisson family and log link function. In GLM response functions, exponentiated coefficients have special meanings. For family Poisson and log link, the meaning of the exponential coefficient refers to the incidence-rate ratio.

The dependent variable in these analyses was unscreened hypertension. The independent variables were lifestyle, the use of substances, diet, anthropometric indicators, being sedentary and comorbidity. This model was adjusted by sex and a differential weight for the frequency of participation by neighborhood. The variables explored in the GLM were selected for their theoretical relevance (figure 1). Some studies show that gaps in hypertension care are disproportionately distributed among different sociodemographic groups, suggesting that hypertension control is significantly lower in younger individuals [15, 22, 29]. Also, women, poor individuals, people with low levels of education, a lifestyle of risk (e.g., unhealthy food, little exercise, use of alcohol), and those living in rural settings were most likely to have untreated and non-controlled high blood pressure or to be unaware of their hypertension [18, 29]. Finally, this ends up in what Dhungana et al [4] hypothesized about the healthcare process. The lack of care in any steps becomes undetected, unconscious, untreated, and uncontrolled hypertension.

This study followed all international standards for research with human subjects and Colombian standard 8430 from 1993 [30]. Participation was voluntary and took informed consent. Prior to data collection, the study was approved through Research Center and Ethical Review Board of the Sabaneta campus of San Martin University (CI-FUSM-2022-001). Before data collection a written consent was informed to the patients. The data collection tool was also entirely confidential. The authors not had access to information that could identify individual participants during or after data collection.

Figure 1.
A theoretical model for the relationship between unscreened hypertension and risk factors in adults over 18 years.
Source: created by the authors.

Results

The prevalence of hypertension was 38.5% (95.0% CI: 32.7, 44.3). Among the 110 participants with hypertensives, the prevalence of undiagnosed hypertension was 50.9% (95.0% CI: 41.1, 60.7) (Figure 2).

Figure 2.
Prevalence of hypertension and unscreened hypertension in adults over 18 years. Sabaneta, 2022.
Source: created by the authors.

The sample demographic characteristics are shown in table 1. Most were female (57.0%) and under 60 (63.9%). Age was distributed between 19 and 91 years (median age = 51.2 years; Interquartile range = 34.3 years; mean age = 50.2 years; SD = 18.8 years). A little more than 80% of those surveyed have their health needs covered through the contributory scheme or as a beneficiary (one of how the salaried population has access to health care in the current Colombian health care system). In the population, 40.2% had completed primary school, and 30.1% had completed high school. As for marital status, the highest proportion were married or in a partnership (45.5%). Regarding socioeconomic status, the highest proportion (58.0%) were low-income. We have not found significant differences between hypertense or normotensive by demographic characteristics (value $p > 0.05$).

Table 1.
Demographic characteristics in adults over 18 years by hypertension. Sabaneta, 2022.

Factor	Normotensive n (%)	Hypertense n (%)	Total	p value[a]
**Sex**				
Female	101 (62.0)	62 (38.0)	163 (57.0)	0.865
Male	75 (61.0)	48 (39.0)	123 (43.0)	
**Age (Years)**				

Factor	Normotensive n (%)	Hypertense n (%)	Total	p value[a]
< 60	120 (65.6)	63 (34.4)	183 (63.9)	0.074
>= 60	56 (54.4)	47 (45.6)	103 (36.1)	
**Social protection in health**				
Beneficiary / Contributor	135 (62.2)	82 (37.8)	217 (84.4)	0.791
Linked / Other	24 (60.0)	16 (40.0)	40 (15.6)	
**Education level**				
No formal education	3 (50.0)	3 (50.0)	6 (2.1)	0.214[b]
Primary school	62 (53.9)	53 (46.1)	115 (40.2)	
High school	57 (66.3)	29 (33.7)	86 (30.1)	
Technical or technological	25 (71.4)	10 (28.6)	35 (12.2)	
University / post-grad	29 (65.9)	15 (34.1)	44 (15.4)	
**Marital status**				
Single	76 (67.9)	36 (32.1)	112 (39.2)	0.151
Married / partnership	77 (59.2)	53 (40.8)	130 (45.5)	
Separated / divorced / widowed	23 (52.3)	21 (47.7)	44 (15.4)	
**Socioeconomic status**				
Low	99 (62.3)	60 (37.7)	159 (58.0)	0.503
Middle	67 (58.3)	48 (41.7)	115 (42.0)	
**Sample**	**176**	**110**	**286**	

a: chi-square test of association

b: Fisher test

Around half of the participants (55.9%) knew about the health benefits plan. Alcohol use prevailed in 60.0% of the participants. Only 38.2% and 50.7% consumed the recommended fruit and vegetables. Of the hypertensive participants, 49.4% also had dyslipidemia, and 42.1% were sedentary. 42.0% of the respondents were overweight. Among hypertensives, the percentage of obese (56.0%) and those participants with abdominal obesity (50.9%) were higher (Table 2). As shown in Table 2, we found significant statistical differences in hypertension according to alcohol use (p=0.043), presence of dyslipidemia (p=0.043), sedentary lifestyle (0.044), BMI (p= 0.001), and abdominal obesity (p=0.001).

Table 2.

Clinical history and lifestyle in adults older than 18 years by hypertension. Sabaneta, 2022.

Factor	Normotensive	Hypertense	Total	p value[a]
	n (%)	n (%)		
**Knowledge of health programs**				
Health benefits plan	99 (67.3)	48 (32.7)	147 (55.9)	0.060
Cardiovascular Program	44 (64.7)	24 (35.3)	68 (25.9)	0.642
**Use of substance**				
Psychoactive	23 (69.7)	10 (30.3)	33 (11.6)	0.298
Tobacco	26 (70.3)	11 (29.7)	37 (12.9)	0.242
Alcohol	114 (66.3)	58 (33.7)	172 (60.1)	0.043
**Food**				
Vegetable oil	162 (61.8)	100 (38.2)	262 (91.6)	0.391[b]
At least 7 fruits a week	63 (58.9)	44 (41.1)	107 (38.2)	0.166
At least 7 vegetables per week	92 (63.9)	52 (36.1)	144 (50.7)	0.227
**Comorbidity**				
Diabetes	16 (44.4)	20 (55.6)	36 (15.9)	0.064
Dyslipidemia	41 (50.6)	40 (49.4)	81 (35.7)	0.043
Heart attack	13 (59.1)	9 (40.9)	22 (7.7)	0.806
**Sedentary**				
Active	61 (70.9)	25 (29.1)	86 (35.1)	0.044
Sedentary	92 (57.9)	67 (42.1)	159 (64.9)	
**BMI**				
Normal				
Overweight	71 (59.2)	49 (40.8)	120 (4.0)	0.001
Obese	22 (44.0)	28 (56.0)	50 (17.5)	

Factor	Normotensive	Hypertense	Total	p value[a]
**Abdominal obesity**	52 (49.1)	54 (50.9)	106 (37.2)	0.001
**Sample**	**176**	**110**	**286**	

a: chi-square test of association

b: Fisher test

BMI: Body Mass Index

Table 3 shows the risk factors for presenting undiagnosed hypertension among adults classified as hypertensive (n=110) according to the parameters applied in this study. We found that adults under 60 years were at a higher risk of developing hypertension and are oblivious to this, compared to adults above this age (aRR= 1.37, 95.0% CI: 1.13, 1.67). People without comorbidities (dyslipidemia or diabetes) and physically active (according to the Perez-Rojas test) presented a higher than 80.0% risk of undiagnosed hypertension compared to people with comorbidities and sedentarism. Finally, it was confirmed that compared to obese people (traditionally more susceptible to hypertension), overweight participants (aRR=1.41, 95.0%: 1.12, 1.78) had a higher risk of having undiagnosed hypertension.

Table 3. A multivariate model of risk factors for the burden of undiagnosed hypertension in adults over 18 years. Sabaneta, 2022.

Factor	cRR	95% CI – cRR	aRR	95% CI – aRR	Stand. Err.	p value
**< 60 years (ref: >60)**	1.27	1.01 - 1.49	1.37	1.13 - 1.67	0.14	0.001
**No previous diagnosis of dyslipidemia or diabetes (ref: Si)**	2.23	1.86 - 2.67	1.81	1.48 - 2.22	0.19	0.000
**Active (Perez Rojas Test) (ref: inactive)**	1.93	1.63 - 2.28	1.84	1.54 - 2.21	0.17	0.000
**BMI (ref: obese)**						
Normal	1.22	0.96 - 1.55	1.22	0.94 - 1.58	0.16	0.130
Overweight	1.50	1.21 - 1.87	1.41	1.12 - 1.78	0.17	0.004

Adjusted by sex - AIC for adjusted model: 1724,901 - AIC for null model: 2230,008 - model goodness-of-fit p=0,000 - BMI: Body Mass Index - cRR: Crude Relative Risk - aRR: Adjusted Relative Risk - Stand. Err: Standard Error

Discussion

The study aimed to estimate undiagnosed hypertension prevalence and associated factors among people older than 18 in a Colombian municipality. We found a prevalence of this pathology of 38.5%. Five in ten hypertensives in this study had undiagnosed HTN. In the current study, younger adults with no chronic disease who were physically active and not obese were more at risk of being unaware of having hypertension.

The prevalence found in this study (38.5%) was higher than the national and world average, 24.0% and 30.0%, respectively [3, 11, 31], and similar in Latin America. A 2019 study, which included 33,276 participants from six Latin American countries, found an HTN prevalence of 44.0%, with the lowest rates in Peru (17.7%) and the highest rates in Brazil (52.5%); moreover, only 58.9% were aware of HTN diagnosis [32].

In terms of the prevalence of undiagnosed hypertension (50.9%), the data differ among studies based on the clinic register or surveillance system, as is the case in the United States with 7.0% undiagnosed HTN [21], and China with 28.8% [33]. However, it was similar in representative and population-based investigations generally conducted outside the Americas, ranging from 50.4% to 69.0% undiagnosed HTN [16, 17, 18, 19]. This was relatively equal to that reported in Malaysia (51.6%) [17] and Bangladesh (50.1%) [19]; while it varied a little in Africa (69.8%) [18], with an almost doubled prevalence in Nepal (34.1%) [4]. Our findings exceed the last reports of undiagnosed hypertension in Latin America (ranging from 24% to 47%) [20], but it was below the report for Peru (67.0%) [22]. It is clear that approximately half of the hypertensive individuals are unaware of their hypertensive status; this is known as the "rule of halves", which also applies to other populations.

The type of sample or source data may explain these variations in the prevalence of hypertension and undiagnosed HTN. Most Latin American nationwide surveys are based on self-reports or no series measurements of BP, and so may have underestimated prevalences [5, 32]. Estimates being population-based are limited [18, 19]. Generally, they are based on secondary data such as clinical registries, epidemiological surveillance data, or national surveys (e.g., demographic and health surveys) [14, 15, 16, 17, 22, 34], designed for different purposes. They may not adequately represent the population at risk.

Other possible reasons for the high prevalence of undiagnosed HTN may include a lack of awareness of routine blood pressure screening. The frequency of blood pressure measurement is related to hypertension awareness, an aspect essential in diagnosing [4, 19]. Clearly, and as shown in this study, the prevalence of undiagnosed hypertension has not decreased in recent decades [12, 20, 22]. The fact that HTN awareness and screening have not progressed during the last decades is a concern. It highlights the ineffectiveness of existing approaches for detecting HTN, although BP measurement is simple and inexpensive. Controlling arterial hypertension continues to be a pending subject in the region [12], and Primary Health Care (PHC) plays an essential role in early detection. Public health programs must target the prevention, detection, treatment, and control of total cardiovascular risk.

Although the entire population should be the target for HTN detection and control in primary health care, our analysis showed that younger people with no chronic disease who were physically active and not obese had a significant risk of undiagnosed HTN, so a particular focus on these subgroups is warranted. Previous investigations found similar factors of risk [18, 22, 33]. This may be explained by the fact that older adults, people with chronic diseases, comorbidity, or risk factors such as obesity or being overweight are more likely to use

healthcare services, increasing the chances of a diagnosis of HTN [13, 18]. In contrast, people with high-practice exercise may be less health conscious and feel less susceptible to chronic diseases [14]. In other studies, [14, 18] found a positive association between high physical activity and undiagnosed HTN.

According to the present study and some research [14, 19, 22, 33], the healthcare need factors inversely associated with undiagnosed HTN included obesity, other chronic diseases, and diabetes. People without other chronic ailments have less likelihood of accessing health services, reducing the probability of having a blood pressure measurement [1, 6]. Finally, the focus of our study suggests early screening and targeting younger adults (under 60 years). Consistent with other authors, it is confirmed that young adults are often unaware of hypertension [18]. Previous research suggested that being 50 or older was associated with a lower prevalence of undiagnosed hypertension [4, 15, 22].

Current evidence suggests that the use of health services in recent months, the use of health insurance, or healthy screening were associated with less undiagnosed HTN [4, 15, 18, 22, 33]. The use of health services helps facilitate the best possible response in the hypertension care cascade [4], which includes hypertension screening, awareness, treatment, and control. However, no information is available in the present study to clarify this relationship.

Evidence shows that current prevention and control strategies are failing. A more compelling, comprehensive, and sustainable approach is urgently needed to reduce the burden of CVD and contribute to increasing the resilience of health systems. PHC is key to balancing the hypertension care cascade [12, 35]. In LMICs countries such as Colombia, there is a lower culture of prevention and screening, and the population generally attends the service when symptoms are present. It is not for nothing that these countries have a 75.0% prevalence of

hypertension [1, 6]. Health models based on the PHC strategy could potentially be a gateway to HTN early detection services. One of the current strategies in the Americas is the implementation of the HEARTS Project [12, 35].

The introduction of programs such as HEARTS, establishes a new model where the management of hypertension and prevention of CVD is no longer centered at the secondary or tertiary level of care, but in the primary care model [12, 35]. It also proposes a training and education model for a PHC team to be better informed about the community, patients, and context and use ways of linking and approaching the population, such as telemonitoring [12, 24, 36]. Still, this model presents challenges to accessing the population that does not demand the service. Therefore, it is necessary to transcend the thresholds of healthcare institutions and be linked with the community and local health perspectives, linking community facilitators and extra-mural strategies. A standardized methodology for participant sampling, BP measurement, and data quality control and analysis is also necessary. Finally, research to develop and implement effective, equitable, and sustainable evidence-based interventions and public health policies worldwide is critical in reducing the global burden of hypertension [6].

The study had several limitations. First, inferring causality was not possible in the cross-sectional design. Second, self-reported data (e.g., diagnosed hypertension) might have incurred recall bias. Third, a family history of hypertension was not included. Fourth, the blood pressure was measured three times in physical examinations in the study, which was different from Hypertension Clinical Practice Guidelines recommending an average of ≥2 readings obtained on ≥2 occasions [24]. Despite these limitations, our study was unique. It used a population-based sample representative of adults throughout the municipality,

comprehensively examined the risk factors associated with undiagnosed hypertension, and provided clues for future hypertension prevention programs.

In conclusion, the prevalence of hypertension and undiagnosed HTN is high and continues to challenge Colombia and the Americas. National and local-level public health interventions are needed to improve hypertension screening, awareness, treatment, and control in Colombia and Latin America. Mass screening targets the most heavily affected population groups, increasing access to quality care services in primary public healthcare and applying communitarian interventions to address the barriers to screening hypertension early.

References

1. Mills K, Stefanescu A, He J. The global epidemiology of hypertension. Nat Rev Nephrol. 2020;16(4):223-37. doi:https://doi.org/10.1038/s41581-019-0244-2

2. Collaborators GCoD. Global, regional, and national age-sex-specific mortality for 282 causes of death in 195 countries and territories, 1980-2017: a systematic analysis for the Global Burden of Disease Study 2017. Lancet. 2018;392(10159):1736-88. doi:10.1016/S0140-6736(18)32203-7

3. Forouzanfar MH, Liu P, Roth GA, Ng M, Biryukov S, Marczak L, et al. Global Burden of Hypertension and Systolic Blood Pressure of at Least 110 to 115 mm Hg, 1990-2015. JAMA. 2017;317(2):165-82. doi:10.1001/jama.2016.19043

4. Dhungana R, Pedisic Z, Dhimal M, Bista B, Courten M. Hypertension screening, awareness, treatment, and control: a study of their prevalence and associated factors in a nationally representative sample fron Nepal. Global Health Action. 2022;15(1):2000092. doi:10.1080/16549716.2021.2000092

5. Geldsetzer P, Manne-Goehler J, Marcus ME, Ebert C, Zhumadilov Z, Wesseh CS, et al. The state of hypertension care in 44 low-income and middle-income countries: a cross-sectional study of nationally representative individual-level data from 1·1 million adults. Lancet. 2019;394(10199):652-62. doi:10.1016/S0140-6736(19)30955-9

6. Mills K, Bundy J, Kelly T, Reed J, Kearney P, Reynolds K, et al. Global Disparities of hypertension prevalence and control: A systematic analysis of population-based studies dfrom 90 countries. Circulation. 2016;134(6):441-50. doi:doi: 10.1161/CIRCULATIONAHA.115.018912

7. (NCD-RisC) NRFC. Worldwide trends in blood pressure from 1975 to 2015: a pooled analysis of 1479 population-based measurement studies with 19·1 million participants. Lancet. 2017;389(10064):37-55. doi:10.1016/S0140-6736(16)31919-5

8. Gaziano TA, Bitton A, Anand S, Weinstein MC, Hypertension ISo. The global cost of nonoptimal blood pressure. J Hypertens. 2009;27(7):1472-7. doi:10.1097/HJH.0b013e32832a9ba3

9. Manrique-Abril FG, Herrera-Amaya GM, Manrique-Abril RA, Beltrán-Morera J. [Costs of a primary health care program for the management of high blood pressure in Colombia]. Rev Salud Publica (Bogota). 2018;20(4):465-71. doi:10.15446/rsap.V20n4.64679

10. Martinez R, Soliz P, Campbell NRC, Lackland DT, Whelton PK, Ordunez P. Association between population hypertension control and ischemic heart disease and stroke mortality in 36 countries of the Americas, 1990-2019: an ecological study. Rev Panam Salud Publica. 2022;46:e143. doi:10.26633/RPSP.2022.143

11. (NCD-RisC) NRFC. Worldwide trends in hypertension prevalence and progress in treatment and control from 1990 to 2019: a pooled analysis of 1201 population-representative studies with 104 million participants. Lancet. 2021;398(10304):957-80. doi:10.1016/S0140-6736(21)01330-1

12. Sosa Liprandi Á, Baranchuk A, López-Santi R, Wyss F, Piskorz D, Puente A, et al. [Control of arterial hypertension: a pending issueControle da hipertensão arterial: um assunto inacabado]. Rev Panam Salud Publica. 2022;46:e147. doi:10.26633/RPSP.2022.147

13. Martinez R, Soliz P, Mujica OJ, Reveiz L, Campbell NRC, Ordunez P. The slowdown in the reduction rate of premature mortality from cardiovascular diseases puts the Americas

at risk of achieving SDG 3.4: A population trend analysis of 37 countries from 1990 to 2017. J Clin Hypertens (Greenwich). 2020;22(8):1296-309. doi:10.1111/jch.13922

14. Hasan MM, Tasnim F, Tariqujjaman M, Ahmed S, Cleary A, Mamun A. Examining the prevalence, correlates and inequalities of undiagnosed hypertension in Nepal: a population-based cross-sectional study. BMJ Open. 2020;10(10):e037592. doi:10.1136/bmjopen-2020-037592

15. Ntenda PAM, El-Meidany WMR, Tiruneh FN, Motsa MPS, Nyirongo J, Chirwa GC, et al. Determinants of self-reported hypertension among women in South Africa: evidence from the population-based survey. Clin Hypertens. 2022;28(1):39. doi:10.1186/s40885-022-00222-5

16. Kothavale A, Puri P, Yadav S. The burden of hypertension and unmet need for hypertension care among men aged 15-54 years: a population-based cross-sectional study in India. J Biosoc Sci. 2022;54(6):1078-99. doi:10.1017/S0021932021000481

17. Lim OW, Yong CC. The Risk Factors for Undiagnosed and Known Hypertension among Malaysians. Malays J Med Sci. 2019;26(5):98-112. doi:10.21315/mjms2019.26.5.9

18. Pengpid S, Peltzer K. Prevalence and associated factors of undiagnosed hypertension among adults in the Central African Republic. Sci Rep. 2022;12(1):19007. doi:10.1038/s41598-022-23868-5

19. Haider MR, Gupta RD. Inequalities in Undiagnosed Hypertension Among Adult Population in Bangladesh: Evidence from a Nationally Representative Survey. High Blood Press Cardiovasc Prev. 2022;29(1):57-64. doi:10.1007/s40292-021-00488-0

20. Hernández-Hernández R, Silva H, Velasco M, Pellegrini F, Macchia A, Escobedo J, et al. Hypertension in seven Latin American cities: the Cardiovascular Risk Factor Multiple

Evaluation in Latin America (CARMELA) study. J Hypertens. 2010;28(1):24-34. doi:10.1097/HJH.0b013e328332c353

21. Park S, Gillespie C, Baumgardner J, Yang Q, Valderrama AL, Fang J, et al. Modeled state-level estimates of hypertension prevalence and undiagnosed hypertension among US adults during 2013-2015. J Clin Hypertens (Greenwich). 2018;20(10):1395-410. doi:10.1111/jch.13388

22. Guerrero-Díaz DV, Hernández-Vásquez A, Montoya-Rivera WC, Rojas-Roque C, Chacón Díaz MA, Bendezu-Quispe G. Undiagnosed hypertension in Peru: analysis of associated factors and socioeconomic inequalities, 2019. Heliyon. 2021;7(7):e07516. doi:10.1016/j.heliyon.2021.e07516

23. The sixth report of the Joint National Committee on prevention, detection, evaluation, and treatment of high blood pressure. Arch Intern Med. 1997;157(21):2413-46. doi:10.1001/archinte.157.21.2413

24. Unger T, Borghi C, Charchar F, Khan N, Poulter N, Prabhakaran D, et al. Clinical Practice Guidelines: 2020 International Society of Hypertension Global Hypertension Practice Guidelines. AHA Journal. 2020;75:1334-57. doi:10.1161/HYPERTENSIONAHA.120.15026

25. U.S. Department of Health and Human Services. The seventh report of the join national committe on prevention, detection, evaluation, and treatment of high blood pressure (JNC 7). 2003. Contract No.: 1206.

26. Organización Mundial de la Salud (OMS). Método progresivo de la OMS para la vigilancia de los factores de riesgo de las enfermedades crónicas (STEPS). OMS; 2008.

27. Pérez A, Suárez R, García G, Espinosa A, Linares D. Test de clasificación de sedentarismo, propuesta y validación estadística. La Habana, Cuba: Universidad de Cienfuegos; 2002.

28. Alfonso-Mora M, Vidarte-Claros J, Vélez-Álvarez C, Sandoval-Cuéllar C. Prevalencia de sedentarismo y factores asociados, en personas de 18 a 60 años en Tunja-Colombia. Rev Fac Med. 2013;61:3-8.

29. Prenissl J, Manne-Goehler J, Jaacks L, et al. Hypertension screening, awareness, treatment, and control in India: a nationally representative cross-sectional study among individuals aged 15 to 49 years. Plos Med. 2019;16(5):e1002801. doi:doi: 10.1371/journal.pmed.1002801

30. República de Colombia, Ministerio de Salud. Resolución 8430 de 1993, "Por la cuál se establecen las normas científicas, técnicas y administrativas para la investigación en salud". 1993.

31. Zurique-Sánchez M, Zurique-Sánchez C, Camacho-López P, Sánchez-Sanabria M, Hernández-Hernández S. Prevalencia de hipertensión arterial en Colombia. Revisión sistemática y metaanálisis. Acta Med Colomb. 2019;44(4):20-33. doi:https://doi.org/10.36104/amc.2019.1293

32. Lamelas P, Diaz R, Orlandini A, Avezum A, Oliveira G, Mattos A, et al. Prevalence, awareness, treatment and control of hypertension in rural and urban communities in Latin American countries. J Hypertens. 2019;37(9):1813-21. doi:10.1097/HJH.0000000000002108

33. Zhou J, Fang S. Association between Undiagnosed Hypertension and Health Factors among Middle-Aged and Elderly Chinese Population. Int J Environ Res Public Health. 2019;16(7). doi:10.3390/ijerph16071214

34. Ahmed S, Tariqujjaman M, Rahman MA, Hasan MZ, Hasan MM. Inequalities in the prevalence of undiagnosed hypertension among Bangladeshi adults: evidence from a nationwide survey. Int J Equity Health. 2019;18(1):33. doi:10.1186/s12939-019-0930-5

35. Ordunez P, Campbell NRC, Giraldo Arcila GP, Angell SY, Lombardi C, Brettler JW, et al. [HEARTS in the Americas: innovations for improving hypertension and cardiovascular disease risk management in primary careHEARTS nas Américas: inovações para melhorar a gestão do risco de hipertensão e de doenças cardiovasculares na atenção primária]. Rev Panam Salud Publica. 2022;46:e197. doi:10.26633/RPSP.2022.197

36. Hammersley V, Parker R, Paterson M, Hanley J, Pinnock H, Padfield P, et al. Telemonitoring at scale for hypertension in primary care: An implementation study. PLos Med. 2020;17(6):1-19. doi:https://doi.org/10.1371/journal.pmed.1003124

Contribution:

JS and AE participated in planning, study design and data collection. AE and LG organized the database. JS and LG participated in data analysis and interpretation. AE and LG wrote the first version of the article. All authors participated in the critical revision, final adjustment of the paper, and approved the final version of this manuscript.

Funding:

This study was funded by the San Martin University Foundation (PY-2020-025).

Conflict of interest:

The authors declare that they have no conflicts of interest.

Figure

Click here to access/download;Figure;Figure1.jpg

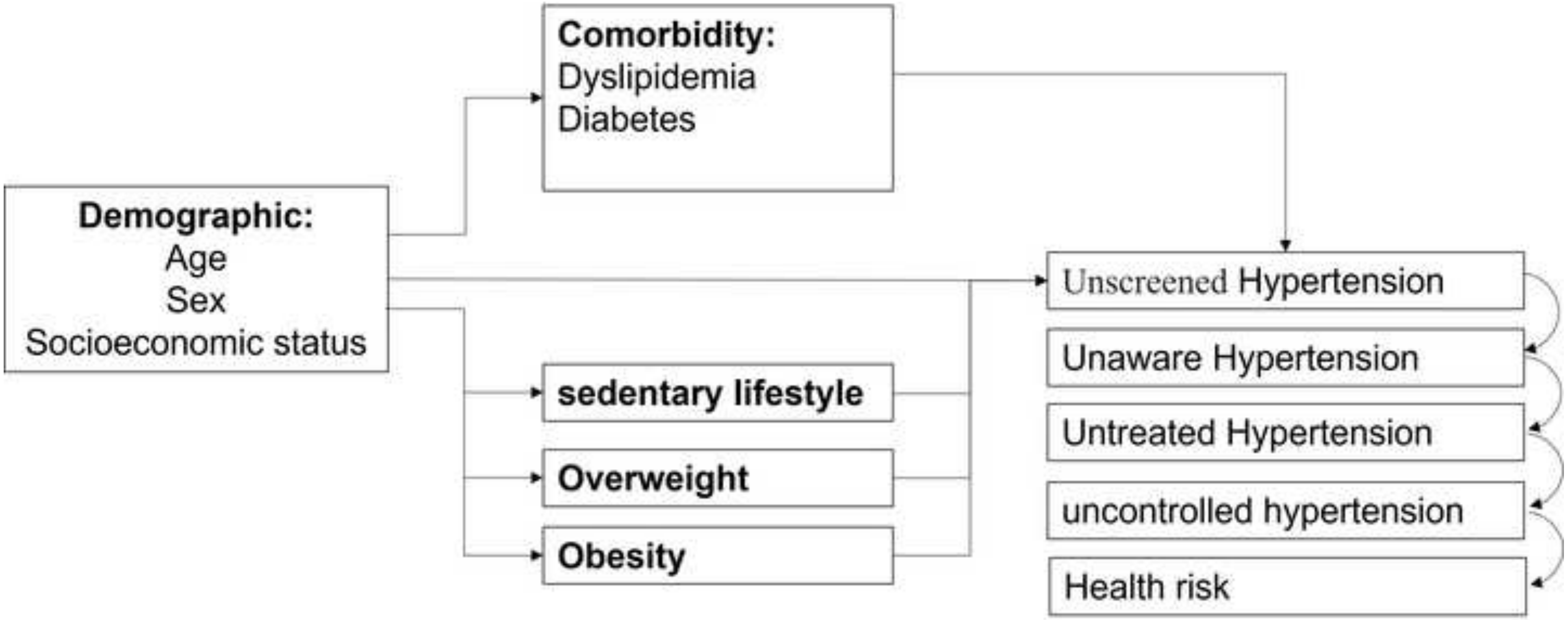

 Click here to access/download;Figure;Figure2.jpg

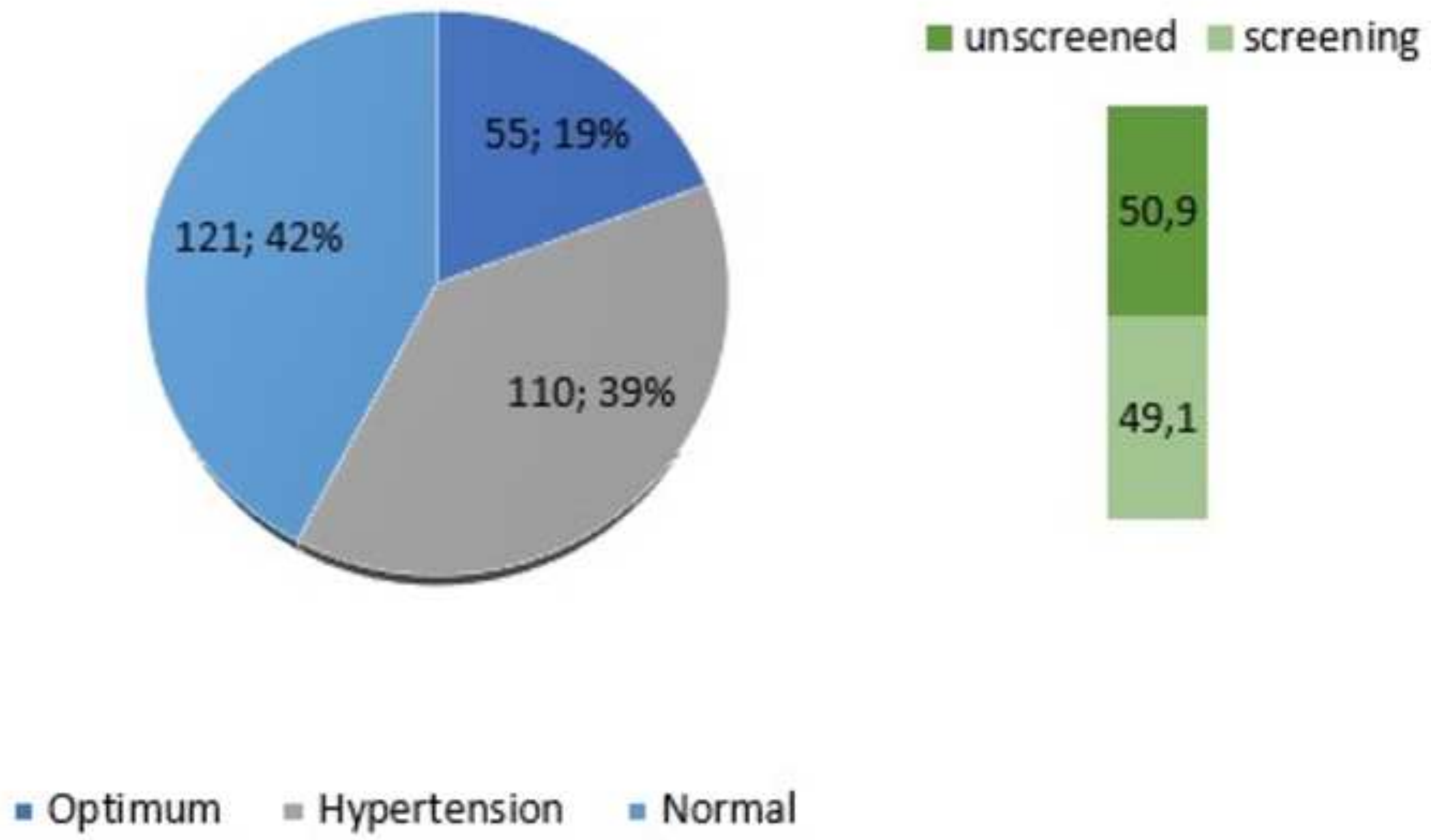

Click here to access/download
Supporting Information
Supplementary 1 - STROBE-checklist.docx
