# Supplementary material for: Burden of undiagnosed hypertension and its associated factors: A challenge for primary health care in urban Colombia
Source: PLoS One. 2023 Nov 28;18(11):e0294177. doi: 10.1371/journal.pone.0294177 (PMC10684019; doi:10.1371/journal.pone.0294177)
Supplement: Attachment — Submitted filename: Response to Reviewers.pdf [file pone.0294177.s004.pdf]

Colombia, August 18th, 2023

Emily Chenette
Editor-in-Chief
PLOS ONE

Dear editor and reviewer,

We want to express our gratitude for your insightful comments, which have been instrumental in enhancing the presentation of findings on this salient public health issue. Kindly find our responses to the comments delineated below. Modifications to the manuscript have been highlighted in blue.

Comments	Reply
The English language needs professional editing throughout the manuscript.	After methodological adjustments, the article was proofread by a native speaker.
The title does not make sense. The word "undiagnostic" in the title should be replaced by "undiagnosed". The title should include the geographical area of the study population. The suggested title may be "Burden of undiagnosed hypertension and its associated factors: A challenge for primary health care in urban Colombia"	The recommendation is implemented by leaving the title as follows: "Burden of undiagnosed hypertension and its associated factors: A challenge for primary health care in urban Colombia" (page 1)
The words "undiagnosed" and "unscreened" were used interchangeably in the manuscript. I suggest using either the word "undiagnosed" or "unscreened" consistently throughout the manuscript. I prefer "undiagnosed".	The word "unscreened" has been removed throughout the document. The word "undiagnosed" is used instead.
In the method section, use the geographical areas from where the study population was selected clearly.	The location of the study is specified in both the abstract and the methodology. "Sabaneta, Colombia"
The authors used words like "incidence" (sentence 168); Risk Ratio (sentence 169);	Analyses were adjusted for the prevalence study. In addition, the proposed multinomial

Comments	Reply
incidence rate ratio (sentence 174) which are wrong for this study as it is a cross-sectional study.	analysis was used, and incidence and relative risks were not used.
The primary dependent variable for this study is undiagnosed hypertension. However, the authors primarily focused on all hypertensions (both diagnosed and undiagnosed) in all analyses. My observation and suggestions are as follows: 38.5% is the overall prevalence of hypertension and half (50.9%) of them are unscreened. So, there are three categories of participants in respect to hypertension: 1) Normotensive, 2) undiagnosed hypertensive, and 3) diagnosed hypertensive. I suggest using a multinomial logistic regression with three category outcomes to identify the associated factors of undiagnosed hypertension with normotensive as the reference category.	We consider the proposed multivariate model to be appropriate. The changes have been implemented and the methodology, results and discussion have been updated.<br><br>The risk factors for normotensive, diagnosed and undiagnosed hypertension were analyzed using multinomial regression. The outcome variable had three categories representing participants with normotensive (reference category), diagnosed and undiagnosed hypertension.
As the number of “no formal education” is low (Table 1), add them to primary education category.	Agreed. Adjustment of frequencies and hypothesis testing is performed. However, all tables have been updated according to the options for the dependent variable: normotensive, diagnosed and undiagnosed HTN.
In table 2, the number of the normal BMI is missing.	We adjusted the frequency of BMI. However, all tables were updated according to the options for the dependent variable: normotensive, diagnosed and undiagnosed HTN.
In sentence 265, the authors mentioned the prevalence of undiagnosed hypertension is 50.9%. I guess this is the prevalence of undiagnosed hypertension among the hypertensive population. Be sure of it.	This is correct. The wording has been improved to make it clear that this is the population with hypertension (page 20).

Comments	Reply
In figure 1, the authors classified the hypertensive status as “unscreened”, “unaware”, “untreated”, “uncontrolled”, and “health risk”. However, these are not shown in the results, although these are important.	We agree with the comment. The model covered the care chain, but this was not explored in depth in the present study, and we do not have data to extend it. Taking this into account, the graph will be updated, leaving only the scope of the results.
Figure 2 is not clear at all. The figure color and legend captions are not related. Either make it clear or delete it.	To make the flow of screening and the corresponding prevalence clearer, the figure has been restructured.

In addition, following the editor's recommendation, we request an update on the project's funding source: San Martin University Foundation (PY-2020-025). This information was not included in the platform's metadata.

Yours sincerely,

Jorge Emilio Salazar Florez
Research center coordinator
San Martin University Foundation, Sabaneta, Colombia

Ángela Patricia Echeverri
Student of medicine
San Martin University Foundation, Sabaneta, Colombia

Luz Stella Giraldo Cardona
Professor
San Martin University Foundation, Sabaneta, Colombia
