# Supplementary material for: Burden of undiagnosed hypertension and its associated factors: A challenge for primary health care in urban Colombia
Source: PLoS One. 2023 Nov 28;18(11):e0294177. doi: 10.1371/journal.pone.0294177 (PMC10684019; doi:10.1371/journal.pone.0294177)
Supplement: Attachment — Submitted filename: Response to Reviewers.pdf [file pone.0294177.s005.pdf]

Colombia, October 20th, 2023

Emily Chenette
Editor-in-Chief
PLOS ONE

Dear Editor Chenette and Reviewers,

We would like to extend our heartfelt gratitude for your constructive comments. They have played a pivotal role in refining our presentation of findings on this significant public health matter. Our responses to your observations are delineated below, and the corresponding changes within the manuscript are highlighted in yellow.

Reviewer 2:

Comments	Reply
Please check the use of HTN (sometimes HNT is used in error).	The acronym has been consistently standardized to "HTN" throughout the document.
Please clarify education level, it sometimes reads as the level of current education, but you maximum level of education gained. Phrases such as 'people in primary school' can be misleading.	We have made it clear that we refer to the highest level of education attained.
It would be nice to add a reference for the population projection (DANE).	The DANE reference has now been incorporated.
Lines 316/317 - I am not sure you mean less health conscious, but maybe they feel they are healthier and probably are healthier so less likley to get checked or screened? I agree with the latter part that they are less susceptible to other diseases.	We meant to indicate that individuals who exercise regularly often feel healthier and thus may be less concerned about regular check-ups. This has been clarified in the text.
Line 371 - be more explicit. Family history was not accounted for as a covariate?	We have made it explicit that family history was not included as a covariate.

Reviewer 3:

Comments	Reply
The introduction is to long, almost 3 pages. I suggest you be more succinct.	We've condensed the introduction for succinctness.
It doesn't make sense for the sentence "Health policymakers might require such empirical evidence to create targeted strategies for controlling HTN both in the country and the world" to be after the objectives. It fits in with the discussion rather than the introduction.	The mentioned sentence has been relocated as suggested.
The sample size is not adequate given that at least 306 participants were supposed to be interviewed (not counting the 10% that is recommended to be added for losses and refusals), and only 286 were interviewed.	We have addressed the sample size limitation in the discussion.
Please check all manuscript and avoid sentences like "hypertensive participants". Change to "participants with hypertension". The same for obese, avoid “were obese or overweight”. Instead, use had obesity of overweight.	Changes have been made to avoid pejorative terms, and the suggested terminologies are adopted.
In general, the manuscript provides interesting results that show a high rate of underdiagnosed cases of hypertension. The failure to reach 100% of the sample calculation needs to be discussed as a limitation. Pejorative terms such as hypertensive people and obese people need to be revised throughout the text. I would also suggest a grammatical review of the English, I believe that some sentences could be rewritten for better understanding.	We appreciate the feedback. The manuscript underwent thorough proofreading by a native English speaker, ensuring clarity and grammatical correctness.

Yours sincerely,

Jorge Emilio Salazar Florez
Research center coordinator
San Martin University Foundation, Sabaneta, Colombia

Ángela Patricia Echeverri
Student of medicine
San Martin University Foundation, Sabaneta, Colombia

Luz Stella Giraldo Cardona
Professor
San Martin University Foundation, Sabaneta, Colombia
