# Supplementary material for: Burden of undiagnosed hypertension and its associated factors: A challenge for primary health care in urban Colombia
Source: PLoS One. 2023 Nov 28;18(11):e0294177. doi: 10.1371/journal.pone.0294177 (PMC10684019; doi:10.1371/journal.pone.0294177)
Supplement: Attachment — Submitted filename: Comments of the manuscript-PONE-D-23-08509_reviewer_dm.docx [file pone.0294177.s003.docx]

Comments of the manuscript (PONE-D-23-08509_reviewer): “BURDEN OF UNDIAGNOSTIC HYPERTENSION ANN ASSOCIATED FACTORS: A CHALLENGE FOR PRIMARY HEALTH CARE”

**Reviewer Comments to Author**

A. MAJOR comments:

Although the manuscript focused on a topic of public health importance, it needs major revision as follows:

1. The English language needs professional editing throughout the manuscript.
2. The title does not make sense. The word “undiagnostic” in the title should be replaced by "undiagnosed". The title should include the geographical area of the study population. Suggested title may be "Burden of undiagnosed hypertension and its associated factors: A challenge for primary health care in urban Colombia"
3. The words “undiagnosed” and “unscreened” were used interchangeably in the manuscript. I suggest using either the word "undiagnosed" or "unscreened" consistently across the manuscript. I prefer “undiagnosed”.
4. In the method section use the geographical areas from where the study population were selected clearly.
5. The authors used words like “incidence” (sentence 168); Risk Ratio (sentence 169); incidence rate ratio (sentence 174) which are wrong for this study as it is a cross-sectional study.
6. The primary dependent variable for this study is undiagnosed hypertension. However, the authors primarily focused on all hypertension (both diagnosed and undiagnosed) in all analyses. My observation and suggestion are as follows:
   i. 38.5% is the overall prevalence of hypertension and half (50.9%) of them are unscreened. So, there are three categories of participants in respect to hypertension: 1) Normotensive, 2) undiagnosed hypertensive, and 3) diagnosed hypertensive. I suggest using a multinomial logistic regression with three category outcome to identify the associated factors of undiagnosed hypertension with normotensive as the reference category.
7. As the number of “no formal education” is low (Table 1), add them with primary education category.
8. In table 2, number of the normal BMI is missing.
9. In sentence 265, the authors mentioned the prevalence of undiagnosed hypertension is 50.9%. I guess this is the prevalence of undiagnosed hypertension among the hypertensive population. Be sure of it.
10. In the figure 1, the authors classified the hypertensive status as “unscreened”, “unaware”, “untreated”, “uncontrolled”, and “health risk”. However, these are not shown in the results, although these are important.
11. Figure 2 is not clear at all. The figure color and legend captions are not related. Either make it clear or delete it.
